# OxymaPure/DIC: An Efficient Reagent for the Synthesis of a Novel Series of 4-[2-(2-Acetylaminophenyl)-2-oxo-acetylamino] Benzoyl Amino Acid Ester Derivatives

**DOI:** 10.3390/molecules181214747

**Published:** 2013-11-28

**Authors:** Ayman El-Faham, Zainab Al Marhoon, Ahmed Abdel-Megeed, Fernando Albericio

**Affiliations:** 1 Department of Chemistry, College of Science, King Saud University, P.O. Box 2455, Riyadh 11451, Saudi Arabia; E-Mail: zanzaki20042000@yahoo.com; 2 Department of Chemistry, Faculty of Science, Alexandria University, P.O. Box 426, Ibrahimia, Alexandria 12321, Egypt; 3 Department of Botany and Microbiology, College of Science, King Saud University, P.O. Box 2455, Riyadh 11451, Saudi Arabia; E-Mail: aamahmoud@ksu.edu.sa; 4 Department of Plant Protection, Faculty of Agriculture, Saba Basha, Alexandria University, Alexandria 12321, Egypt; 5 Institute for Research in Biomedicine (IRB), Barcelona Science Park, Baldiri Reixac 10, Barcelona 08028, Spain; 6 CIBER-BBN, Networking Centre on Bioengineering, Biomaterials and Nanomedicine, Barcelona Science Park, Baldiri Reixac 10-12, Barcelona 08028, Spain; 7 Department of Organic Chemistry, University of Barcelona, Martí i Franqués 1-11, Barcelona 08028, Spain; 8 School of Chemistry & Physics, University of KwaZulu-Natal, Durban 4001, South Africa

**Keywords:** *N*-acetylisatin, 4-aminobenzoic acid, amino acid esters, DIC, OxymaPure, α-ketoamide

## Abstract

OxymaPure (ethyl 2-cyano-2-(hydroxyimino)acetate) was tested as an additive for use in the carbodiimide (DIC) approach for the synthesis of a novel series of α-ketoamide derivatives (4-[2-(2-acetylaminophenyl)-2-oxo-acetylamino]benzoyl amino acid ester derivatives). OxymaPure showed clear superiority to HOBt/DIC or carbodiimide alone in terms of purity and yield. The title compounds were synthesized via the ring opening of *N*-acylisatin. First, *N*-acetylisatin was reacted with 4-aminobenzoic acid under conventional heating as well as microwave irradiation to afford 4-(2-(2-acetamidophenyl)-2-oxoacetamido)benzoic acid. This α-ketoamide was coupled to different amino acid esters using OxymaPure/DIC as a coupling reagent to afford 4-[2-(2-acetylaminophenyl)-2-oxo-acetylamino]benzoyl amino acid ester derivatives in excellent yield and purity. The synthesized compounds were characterized using FT-IR, NMR, and elemental analysis.

## 1. Introduction

**Scheme 1:**
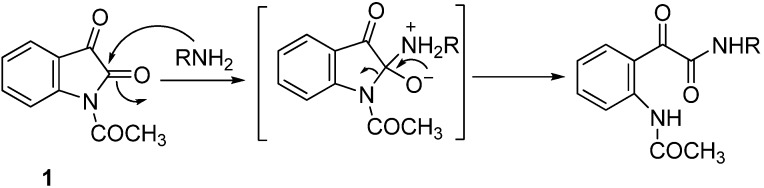
General mechanism for the reaction of *N*-acylisatin (**1**) with amines.

α-Ketoamides are compounds of interest in organic chemistry and are present in many active pharmaceutical compounds [1,2,3,4,5,6]. Parallel with the application of the α-ketoamide moiety in medicinal chemistry, numerous synthetic methods have been described [7,8,9,10,11,12,13,14,15,16,17,18,19,20,21]. Several authors have demonstrated that the synthesis of the α-ketoamide fragment can be achieved by ring opening of *N*-acetylisatin (**1**) by the attack of an amine at C2-carbonyl group of *N*-acetylisatin (Scheme 1) [22,23,24,25,26,27]. Recently, Cheah *et al.* [28,29] reported the reaction of *N*-acetylisatin with L-α-amino acid esters as a novel class of *N*-glyoxylamide peptide mimics.

Here we present the use of OxymaPure/DIC as a coupling reagent for the synthesis of a novel class of α-ketoamide derivatives (4-[2-(2-acetylaminophenyl)-2-oxo-acetylamino]benzoyl amino acid esters).

## 2. Results and Discussion

**Scheme 2:**
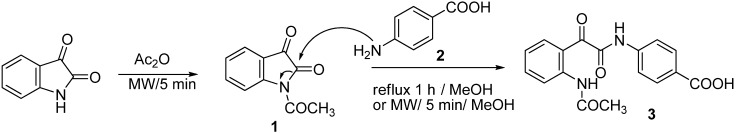
Synthesis and reaction of *N*-acylisatin (**1**) with 4-aminobenzoic acid (**2**).

1-Acetylindoline-2,3-dione (*N*-acetylisatin, **1**) was initially prepared by reaction of isatin with acetic anhydride, using conventional heating under the same conditions as those described in the literature [30,31]. However, we demonstrate that the use of a microwave irradiation, using a multimode reactor (Synthos 3000, Anton Paar GmbH, Graz, Austria, 1,400 W maximum magnetron, method B; Experimental section), renders **1** from isatin and acetic anhydride (Scheme 2) in excellent yield in less reaction time and higher purity than the conventional method, as observed from spectral data. This observation is consistent with data in the literature [30].

*N*-Acetylisatin (**1**) was then reacted with the poor nucleophile, 4-aminobenzoic acid (**2**) using conventional heating for 1 h in methanol as a solvent to afford the product **3** (Scheme 2). The IR and NMR spectral analysis of the product revealed that the reaction proceeded through the ring opening to afford the α-ketoamide derivative **3** and not the Schiff base derivative, in contrast to what was reported in the reaction of bromo-*N*-acetylisatin with aminobenzoic acid. [32,33] The IR spectra of **3** showed three characteristic peaks at 3270, 1679, and 1601 cm^−1^, corresponding to the CO*OH*, the α-ketoamide (COCONH), and NHCOCH_3_, respectively. The ^1^H-NMR of **3** agreed well with the structure, showing eight distinct resonance peaks located at δ 1.99 (s, 3H, COCH_3_), 7.27–7.730 (m, 2H, Ar), 7.62–7.68 (m, 2H, Ar), 7.90 (d, 2H, Ar), 7.90 (d, 2H, Ar), 10.55 (s, 1H, NH), 10.99 (s, 1H, NH), and 13.00 (brs, 1H, COOH). These peaks were assigned to the acetyl group, aromatic proton (isatin), aromatic proton (4-aminobenzoic acid), two NHs, and COOH respectively. The ^13^C-NMR of **3** also confirmed the structure, showing the characteristic signals at δ 161.0, 166.1, 168.2, and 188.1 corresponding to the two amide groups, one carbonyl of the carboxyl group, and the α-ketoamide group respectively, along with the rest of the expected carbon signals of the compound.

The same reaction was repeated under microwave irradiation using a multimode reactor (Anton Paar GmbH Synthos 3000, 1,400 W maximum magnetron, method B in the Experimental section) to afford product **3** (Scheme 2) in less reaction time and high purity as shown by its spectral data. The IR and NMR spectra of **3** proved its structure and were in agreement with the product obtained by conventional heating.

**Scheme 3:**
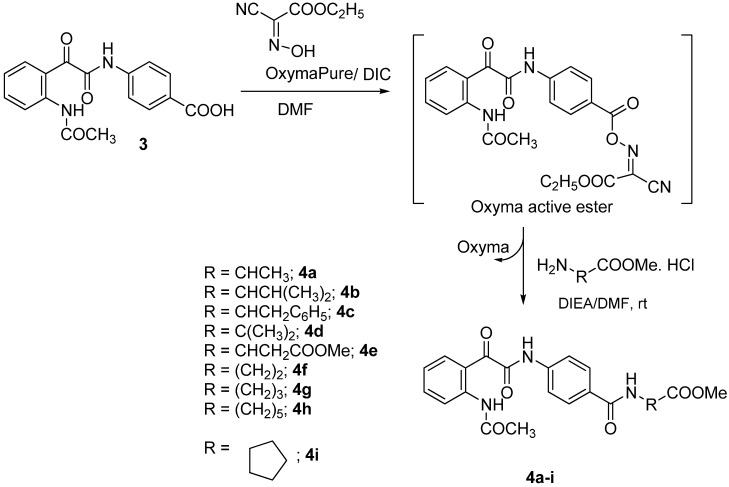
Synthesis 4-[2-(2-acetylaminophenyl)-2-oxo-acetylamino]benzoyl amino acid ester derivatives **4a**–**i**.

Recently, OxymaPure (ethyl 2-cyano-2-(hydroxyimino)acetate, Scheme 3) was used as an additive for peptide synthesis in combination with carbodiimides [34]. It displayed an appropriate balance of availability and ease of handling. In addition it was safer than HOBt and showed clear superiority in terms of coupling efficiency [34,35,36,37,38,39,40,41,42].

Here we tested OxymaPure as an additive for the coupling of compound **3** to H-Ala-OMe·HCl. In a general experiment, **3** was preactivated with OxymaPure/DIC for 5 min in DMF to generate the corresponding active ester (Scheme 3), which reacted directly with H-Ala-OMe·HCl in the presence of 1 equiv. of DIEA at 0 °C for 1 h and then at r.t overnight. After workup and removal of the solvent, product **4a** was obtained as a white solid in 88% yield (Scheme 3).

The reaction of **3** with H-Ala-OMe was then repeated using various coupling reagents (Table 1). The best results were obtained using OxymaPure/DIC; OxymaPure showed clear superiority to HOBt/DIC and carbodiimide alone in terms of yield and purity. DCC consistently showed some impurities from dicyclohexylurea (DCU) as observed from NMR spectra.

**Table 1:** Reaction of **3** with H-Ala-OMe.HCl using various coupling conditions.

Coupling Condition	Yield (%)	Mp (°C)
DIC/Oxyma	88	174–176
DCC/Oxyma *	82	168–172
DIC/HOBt	72	172–175
DCC/HOBt *	70	170–174
DIC	60	170–173
DCC *	60	168–173

* NMR showed impurities corresponding to the dicyclohexylurea byproduct.

The IR spectrum of **4a** showed four characteristic peaks at 3288, 1741, 1672, and 1607 cm^−1^, corresponding to the NH, CO-ester, α-ketoamide and CO-amide, respectively. The ^1^H-NMR showed a doublet peak at δ 1.41, which was related to the CH_3_ for the alanine unit, two singlet peaks at δ 2.00 and 3.65 for the acetyl and the methyl ester, and three singlet peaks at δ 8.73, 10.55, 10.92 for three NHs, respectively. The ^13^C-NMR also confirmed the structure of **4a**, showing signals at δ 17.3 (CHCH_3_), 24.2 (NCOCH_3_), 49.9 (COOCH_3_), 52.1 (CH-NH), 162.3, 166.2, and 169.5 (for three CONHs), 173.8 (COOCH_3_), and 190.0 (COCO), along with the remaining carbon residues related to **4a**.

Several amino acid esters were prepared following the reported method [43,44] and coupled with **3** using OxymaPure/DIC under the same conditions used for the coupling of H-Ala-OMe. This approach afforded products **4a**–**i** in excellent yield and purity (Scheme 3, Table 2). The structures of all the compounds synthesized were confirmed by IR, NMR (^1^H-NMR and ^13^C-NMR) and elemental analysis.

**Table 2:** Yield (%), Mp (°C), and Elemental analysis of **4a**–**i**.

Compd. No	Yield (%)	Mp (°C)	Elemental Analysis Calcd. (Found)		
			C	H	N
**4a**	88	174–176	61.31 (61.60)	5.14 (5.34)	10.21 (10.00)
**4b**	81	178–180	62.86 (63.02)	5.73 (5.96)	9.56 (9.581)
**4c**	83	168–170	66.52 (66.67)	5.17 (5.24)	8.62 (8.88)
**4d**	76	216–218	62.11 (62.38)	5.45 (5.66)	9.88 (10.07)
**4e**	82	154–156	66.52 (66.33)	5.17 (5.26)	8.62( 8.90)
**4f**	88	154–156	61.31 (61.09)	5.14 (5.23)	10.21 (10.48)
**4g**	86	118–120	62.11 (62.37)	5.45 (5.67)	9.88 (10.04)
**4h**	83	180–182	63.56 (63.38)	6.00 (6.13)	9.27 (9.53)
**4i**	78	238–240	63.85 (64.06)	5.58 (5.65)	9.31 (9.04)

## 3. Experimental

### 3.1. General

The solvents used were of HPLC reagent grade. Melting points were determined with a Mel-Temp apparatus and are uncorrected. Fourier transform infrared spectroscopy (FTIR) spectra was recorded on Nicolet 560 spectrometer. Nuclear magnetic resonance spectra (^1^H-NMR and ^13^C-NMR spectra) were recorded on a JOEL 400 MHz spectrometer with chemical shift values reported in δ units (ppm) relative to an internal standard. The microwave irradiation used a multimode reactor (Synthos 3000, Anton Paar GmbH, and 1,400 W maximum magnetron). Elemental analyses were performed on Perkin-Elmer 2400 elemental analyzer, and the values found were within ±0.3% of the theoretical values. Follow-up of the reactions and checks of the purity of the compounds were done by TLC on silica gel-protected aluminum sheets (Type 60 GF254, Merck) and the spots were detected by exposure to a UV-lamp at λ 254 nm for a few seconds. The compounds were named using ChemDraw Ultra version 11, Cambridge Soft Corporation (Cambridge, MA, USA).

### 3.2. Synthesis of 1-Acetyl-1H-indole-2,3-dione (N-acetylisatin) (**1**)

Conventional method (**A**): A mixture of isatin (0.01 M) and acetic anhydride (5 mL) was refluxed for 5 h. After cooling to r. t., it was left to stand overnight. The precipitate was collected, washed with 96% ethanol and air-dried.

Microwave method (**B**): A multimode reactor (Anton Paar GmbH Synthos 3000, 1,400 W maximum magnetron) was used. The initial step was conducted with a 2-Teflon vessels rotor (MF 100). Isatin (5 mmol) was suspended in acetic anhydride (10 mL) and the reaction was processed by heating the vessels for 5 min. at 80 °C and holding it at the same temperature for 5 min (under 0.2/s bar pressure, 400 W). Cooling was accomplished by a fan (for 5 min) and the desired product was obtained as a yellow needle in excellent yield without further recrystallization. The spectral data were in accordance with the data reported in the literature [30].

*1-Acetyl-1H-indole-2,3-dione (N-acetylisatin)* (**1**) The product was obtained as green crystals, mp: 146–148 °C; yields: 79% (method A); 95% (method B) (lit. [23] 141 °C, yield 97%; lit. [30] 137–139 °C, yield 70%). ^1^H-NMR (CDCl_3_) δ (ppm): 2.69 (s, 3H, COCH_3_), 7.31 (t, *J* = 8.04 Hz, 1H), 7.69 (t, *J* = 7.32 Hz,1H), 7.73 (d, *J* = 7.32 Hz, 1H), 8.36 (d, *J* = 8.08 Hz,1H); ^13^C-NMR (CDCl_3_) δ (ppm): 26.5 (COCH_3_), 118.3, 119.2, 125.3, 126.2, 139.0, 148.6, 158.0 (CO), 169.8 (CO), 180.2 (CO).

### 3.3. Synthesis of 4-(2-(2-Acetamidophenyl)-2-oxoacetamido)benzoic Acid (**3**)

Conventional method (**A**): A mixture of *N*-Acetyl isatin (0.01 M) and 4-amino-benzoic acid (0.01 M) in absolute methanol (20 mL) was refluxed for 1 h in the presence of 2–3 drops of glacial acetic acid. After cooling, was filtered and recrystallized from ethanol to afford the product in 96% yield.

Microwave method (**B**): A multimode reactor (Synthos 3000 Aton Paar, GmbH, 1400 W maximum magnetron) was used. The initial step was conducted with 2-Teflon vessels rotor (MF 100) that allows the reaction to be processed under the same conditions. *N-*acetylisatin and 4-aminobenzoic acid were mixed in methanol as a solvent in the presence or absence of glacial acetic acid (2–3 drops). The individual vessels were placed in the corresponding rotor, and finally the rotor was closed with a protective hood. The vessels were heated for 2 min. at 80 °C and held at the same temperature for another 2 min (~2 bar pressure, 400 W). Cooling was accomplished by a fan (5 min). The final product was washed with cold methanol, and then dried under vacuum to afford the product in a pure state as observed from spectral analysis.

The product was obtained as a pale yellow powder, mp: 238–240 °C; yield (57% method A); (86% method B). ^1^H-NMR (DMSO-*d_6_*) δ (ppm): 1.99 (s, 3H, COCH_3_), 7.25–7.30 (m, 2H), 7.62–7.68 (m, 2H), 7.90 (d, *J* = 8.79 Hz, 2H), 7.95 (d, *J* = 8.43 Hz, 2H), 10.55(s, 1H, NH), 10.99 (s, 1H, NH), 13.00 (brs, 1H, COOH); ^13^C-NMR (DMSO-*d6*) δ (ppm): 22.8 (NHCOCH_3_), 118.7, 121.0, 123.1, 124.3, 125.4, 129.6, 130.3, 132.9, 136.8, 141.2, 161.00 (CONH), 166.1(CONH), 168.2(COOH), 188.1 (COCO). IR (cm^−1^): 3270 (COOH), 1679, 1601 (C=O). Anal. Calcd. for C_17_H_14_N_2_O_5_: C, 62.57; H, 4.32; N, 8.59. Found: C, 62.80; H, 4.21; N, 8.33.

### 3.4. General Method for the Synthesis of Amino Acid Esters

Thionyl chloride (10 mL) was slowly added to a cold suspension solution of the appropriate amino acid (50 mmol) in methanol (50 mL) at 0 °C. The reaction mixture was stirred for 8–10 h and then concentrated on a rotary evaporator. The white precipitate formed was washed with anhydrous ether and then dried under vacuum. All data agreed with the reported data [43,44].

*Methyl 1-aminocyclopentane carboxylate HCl.* White powder, mp: 204–206 °C, yield 87%. ^1^H-NMR (DMSO-*d_6_*) δ (ppm): 1.62–1.72 (m, 2H, CH_2_CH_2_CH_2_), 1.81–1.91 (m, 2H, HNCH_2_CH_2_CH_2_), 1.94–1.96 (m, 2H, CH_2_CH_2_CO), 2.07–2.14 (m, 2H, CH_2_CH_2_NH), 3.73 (s, 3H, COOCH_3_), 8.81(brs, 2H, NH_2_). ^13^C-NMR (DMSO-*d_6_*) δ (ppm): 24.3, 35.2, 52.4 (COOCH_3_), 63.4, 171.8(COOCH_3_).

*Methyl 3-aminopropionate HCl.* White powder, mp: 90–92 °C, yield 85%. ^1^H-NMR (DMSO-*d_6_*) δ (ppm): 2.72 (t, *J* = 7.35 Hz, 2H, NH_2_CH_2_CH_2_CO), 2.98 (m, 2H, NH_2_CH_2_CH_2_CO), 3.62 (s, 3H, COOCH_3_), 8.22 (brs, 2H, NH_2_).

*Methyl 4-aminobutanoate HCl.* White powder, mp: 104–106 °C, yield 87%. ^1^H-NMR (DMSO-*d_6_*) δ (ppm): 1.80 (m, 2H, NH_2_CH_2_CH_2_CH_2_CO), 2.43 (t, *J* = 7.0 Hz, 2H, NH_2_CH_2_CH_2_CH_2_CO), 2.78 (m, 2H, NH_2_CH_2_CH_2_CH_2_CO), 3.59 (s, 3H, COOCH_3_), 8.16 (brs, 2H, NH_2_).

*Methyl 6-aminohexanoate HCl.* White powder, mp: 104–106 °C, yield 90%. ^1^H-NMR (DMSO-*d_6_*) δ (ppm): 1.26–1.31 (m, 2H, NH_2_CH_2_CH_2_CH_2_CH_2_CH_2_CO), 1.49–1.57 (m, 4H, NH_2_CH_2_CH_2_CH_2_CH_2_CH_2_CO), 2.29 (t, *J* = 7.30 Hz, 2H, NH_2_CH_2_CH_2_CH_2_CH_2_CH_2_CO), 2.71 (m, 2H, NH_2_CH_2_CH_2_CH_2_CH_2_CH_2_CO), 3.57 (s, 3H, COOCH_3_), 8.08 (brs, 2H, NH_2_).

*Dimethyl 2-aminosuccinate HCl.* White powder, mp: 110–112 °C, yield 82%. ^1^H-NMR (DMSO-*d_6_*) δ (ppm): 3.03 (q, *J* = 4.0 Hz, 2H, COCH_2_CHNH_2_), 3.64 (s, 3H, COOCH_3_), 3.72 (s, 3H, COOCH_3_), 4.33 (m, 1H, CH_2_CHNH_2_), 8.75 (brs, 2H, NH_2_).

### 3.5. General Procedure for the Synthesis of **4a**–**i**

Acid **3** (1 mmol), Oxyma (1 mmol), and DIC (1 mmol) were mixed in DMF (5 mL) at 0 °C. The reaction mixture was stirred for 5 min at 0 °C to preactivate the acid and generate the active ester, and then DIEA (1 mmol) followed by amino acid ester (1 mmol) were added. The reaction mixture was stirred at 0 °C for 1 h and at room temperature overnight. The mixture was then diluted with ethyl acetate (50 mL) and then extracted with 1 N HCl (2 × 10 mL), 10% NaHCO_3_ (2 × 10 mL), and saturated NaCl (2 × 10 mL). The organic phase was dried over anhydrous MgSO_4_, filtered, and the solvent was removed under vacuum. The residue was recrystallized from dichloromethane-hexane to afford the pure product.

*Methyl 2-(4-(2-(2-acetamidophenyl)-2-oxoacetamido)benzamido)propanoate* (**4a**). White powder, mp: 174–176 °C, yield 88%. ^1^H-NMR (DMSO-*d_6_*) δ (ppm): 1.41 (d, *J* = 7.32 Hz, 3H, CHCH_3_), 2.00 (s, 3H, COCH_3_), 3.65 (s, 3H, COOCH_3_), 4.48 (m, 1H, NHC*H*CH_3_), 7.45 (t, *J* = 7.2 Hz, 1H), 7.64 (d, *J* = 4.4 Hz, 1H), 7.68 (d, *J* = 7.36 Hz, 2H), 7.89 (m, 4H, Ar), 8.73(s, 1H, NH), 10.55(s, 1H, NH), 10.92 (s, 1H, NH); ^13^C-NMR (DMSO-*d_6_*) δ (ppm): 17.3 (NHCHCH_3_), 24.2 (COCH_3_), 49.9 (COOCH_3_), 52.1 (NHCHCH_3_), 120.0, 122.4, 124.4, 126.4, 128.9, 129.0, 131.6, 134.21, 139.4, 142.2, 162.3 (CONH), 166.2 (CONH), 169.5 (CONH), 173.8 (COOCH_3_), 190.0 (COCO). IR (cm^−1^): 3288, 3124 (NH), 1741, 1672, 1607, 1536 (C=O). Anal. Cacld for C_21_H_21_N_3_O_6_: C, 61.31; H, 5.14; N, 10.21. Found: C, 61.60; H, 5.34; N, 10.00.

*Methyl 2-(4-(2-(2-acetamidophenyl)-2-oxoacetamido)benzamido)-3-methylbutanoate* (**4b**). White powder, mp: 178–180 °C, yield 81%. ^1^H-NMR (CDCl_3_) δ (ppm): 1.00 (t, *J* = 8.60 Hz, 6H, CH (CH_3_)_2_), 1.59 (m, 1H, CHCH(CH_3_)_2_), 2.27 (s, 3H, COCH_3_), 3.78 (s, 3H, COOCH_3_), 4.78 (m, 1H, CHCHNH), 6.61 (d, *J* = 8.08 Hz, 1H), 7.17 (t, *J* = 8.08 Hz, 1H), 7.64 (t, *J* = 8.08 Hz, 1H), 7.78 (d, *J* = 8.08 Hz, 1H), 7.86 (d, *J* = 8.08 Hz, 2H), 8.50 (d, *J* = 8.08 Hz, 1H) 8.64 (d, *J* = 8.08 Hz, 1H), 8.99 (s, 1H, NH), 10.79 (s, 1H, NH); ^13^C-NMR (CDCl_3_) δ (ppm): 18.1, 19.1(CHCH(CH_3_)_2_), 25.0 (COCH_3_), 31.0 (CHCH(CH_3_)_2_), 52.4 (COOCH_3_),57.6 (CHCH(CH_3_)_2_), 119.4, 119.8, 120.9, 122.8, 128.4, 130.6, 134.3, 136.8, 140.0, 142.1,160.6 (CONH), 166.5 (CONH), 169.4 (CONH), 172.8 (COOCH_3_), 190.8 (COCO). IR (cm^−1^): 3293 (NH), 1747, 1679, 1634, 1608, 1526 (C=O). Anal. Cacld. for C_23_H_25_N_3_O6_3_: C, 62.86; H, 5.73; N, 9.56. Found: C, 63.02; H, 5.96; N, 9.81.

*Methyl 2-(4-(2-(2-acetamidophenyl)-2-oxoacetamido)benzamido)-3-phenylpropanoate* (**4c**). White powder, mp: 168–170 °C, yield 83%. ^1^H-NMR (CDCl_3_) δ (ppm): 2.24 (s, 3H, COCH_3_), 3.25 (m, 2H, CHCH_2_C_6_H_5_), 3.80 (s, 3H, COOCH_3_), 5.08 (m, 1H, CHCH_2_C_6_H_5_), 6.56 (d, *J* = 7.36 Hz, 1H), 7.12 (d, *J* = 6.6 Hz, 1H), 7.15–7.29 (m, 5H, CHCH_2_C_6_H_5_), 7.65 (t, *J* = 6. 6 Hz, 1H), 7.76 (m, 4H, NHC_6_H_4_CO), 8.47 (d, *J* = 8.08 Hz, 1H), 8.63 (d, *J* = 8.08 Hz, 1H), 9.01(s, 1H, NH), 10.79 (s, 1H, NH); ^13^C-NMR (CDCl_3_) δ (ppm): 25.0 (COCH_3_), 38.0 (CHCH_2_C_6_H_5_), 49.2 (COOCH_3_), 52.6 (CHCH_2_C_6_H_5_), 118.8, 119.8, 121.0, 122.8, 127.3, 128.39, 128.7, 129.4, 130.5, 134.4, 135.9, 136.9, 139.9, 142.1, 160.3 (CONH), 166.0 (CONH), 169.4 (CONH), 172.1 (COOCH_3_), 190.6 (COCO). IR (cm^−1^): 3250, 3116 (NH), 1750, 1679, 1635, 1608, 1523 (C=O). Anal. Calcd for C_27_H_25_N_3_O_6_: C, 66.52; H, 5.17; N, 8.62. Found: C, 66.67; H, 5.24; N, 8.88.

*Methyl 2-(4-(2-(2-acetamidophenyl)-2-oxoacetamido)benzamido)-2-methylpropanoate* (**4d**). White powder, mp: 212–218 °C, yield 76%. ^1^H-NMR (DMSO-*d_6_*) δ (ppm): 1.46 (s, 6H, HNC(CH_3_)_2_), 1.99 (s, 3H, COCH_3_), 3.58 (s, 3H, COOCH_3_), 7.63–7.87 (m, 8H, NHC_6_H_4_COCONHC_6_H_4_CO), 8.54 (s, 1H, NH), 10.56 (s, 1H, NH), 10.90 (s, 1H, NH); ^13^C-NMR (DMSO-*d_6_*) δ (ppm): 25.6 (COCH_3_), 40.5 (HNC(CH_3_)_2_), 52.4 (COOCH_3_), 56.1 HNC(CH_3_)_2_, 119.8, 121.3, 124.5, 126.4, 129.0, 130.0, 131.5, 134.2, 138.1, 142.20,158.0 (CONH), 165.9 (CONH), 169.5(CONH), 175.1 (COOCH_3_), 189.5 (COCO). IR (cm^−1^): 3428 (NH), 1749, 1680, 1637, 1525 (C=O). Anal. Calcd. for C_22_H_23_N_3_O_6_: C, 62.11; H, 5.45; N,9.88. Found: C, 62.38; H, 5.66; N, 10.07.

*Dimethyl 2-(4-(2-(2-acetamidophenyl)-2-oxoacetamido)benzamido)*succinate (**4e**). White powder, mp: 154–156 °C, yield 82%. ^1^H-NMR (DMSO-*d_6_*) δ (ppm): 1.99 (s, 3H, COCH3), 2.85–2.98 (m, 2H, CHCH_2_COOCH_3_), 3.62 (s, 3H, COOCH_3_), 3.65 (s, 3H, COOCH_3_), 4.83 (m, 1H, HNCHCH_2_), 7.28–7.87 (m, 8H, NHC_6_H_4_COCONHC_6_H_4_CO), 8.86 (d, 1H, NH), 10.54 (s, 1H, NH), 10.93 (s, 1H, NH); ^13^C-NMR (DMSO-*d_6_*) δ (ppm): 23.9 (COCH_3_), 36.0 (NHCHCH_2_CO), 49.8 (NHCHCH_2_CO), 52.3 (COOCH_3_), 52.8 (COOCH_3_), 119.9, 122.4, 124.4, 125.7, 128.9, 129.5, 131.55, 134.2, 138.1, 141.5, 162.3 (CONH), 166.2 (CONH), 169.5 (CONH), 171.1(COOCH_3_), 171.9 (COOCH_3_), 189.5 (COCO). IR (cm^−1^): 3295 (NH), 1748, 1667, 1608, 1526 (C=O). Anal. Calcd. for C_27_H_25_N_3_O_6_: C, 66.52; H, 5.17; N, 8.62. Found: C, 66.33; H, 5.26; N, 8.90.

*Methyl 3-(4-(2-(2-acetamidophenyl)-2-oxoacetamido)benzamido)propanoate* (**4f**). White powder, mp: 154–156 °C, yield 88%. ^1^H-NMR (DMSO-*d_6_*) δ (ppm): 1.99 (s, 3H, COCH_3_), 2.60 (t, *J* = 6.60 Hz, 2H, NHCH_2_CH_2_CO), 3.49 (q, 2H, NHCH_2_CH_2_CO), 3.61 (s, 3H, COOCH_3_), 7.29–7.85 (m, 8H, NHC_6_H_4_COCONH C_6_H_4_CO), 8.51 (s, 1H, NH), 10.55 (s, 1H, NH), 10.90 (s, 1H, NH); ^13^C-NMR (DMSO-*d_6_*) δ (ppm): 23.9 (COCH_3_), 34.1 (NHCH_2_CH_2_CO), 36.1(NHCH_2_CH_2_CO), 52.0 (COOCH_3_), 119.9, 122.4, 124.4, 125.6, 128.6, 130.4, 131.6, 134.3, 138.2, 141.1, 162.4 (CONH), 166.3 (CONH), 169.5 (CONH), 172.4 (COOCH_3_), 189.6 (COCO). IR (cm^−1^): 3295 (NH), 1742, 1666, 1635, 1608, 1527 (C=O). Anal. Calcd. for C_21_H_21_N_3_O_6_: C, 61.31; H, 5.14; N, 10.21. Found: C, 61.09; H, 5.23; N, 10.48.

*Methyl 4-(4-(2-(2-acetamidophenyl)-2-oxoacetamido)benzamido)butanoate* (**4g**). White powder, mp: 118–120 °C, yield 86%. ^1^H-NMR (DMSO-*d_6_*) δ (ppm): 1.77 (m, 2H, NHCH_2_CH_2_CH_2_CO), 1.99 (s, 3H, COCH_3_), 2.37 (t, *J* = 7.32 Hz, 2H, NHCH_2_CH_2_CH_2_CO), 3.27 (q, 2H, NHCH_2_CH_2_CH_2_CO), 3.58 (s, 3H, COOCH_3_), 7.28 (t, *J* = 7.3, 2.2 Hz, 1H), 7.64–7.85 (m, 7H, Ar), 8.42 (brs, 1H, NH), 10.55 (s, 1H, NH), 10.89 (s, 1H, NH); ^13^C-NMR (DMSO-*d_6_*) δ (ppm): 23.9 (NHCH_2_CH_2_CH_2_CO), 24.2 (COCH_3_), 31.4 (NHCH_2_CH_2_CH_2_CO), 41.6 (NHCH_2_CH_2_CH_2_CO), 51.8 (COOCH_3_), 119.9, 122.4, 124.4, 125.6, 128.6, 130.7, 131.6, 134.3, 138.7, 141.0, 162.3 (CONH), 166.2 (CONH), 169.5 (CONH), 173.7 (COOCH_3_), 189.7 (COCO). IR (cm^−1^): 3343 (NH), 1738, 1661, 1630, 1600, 1527 (C=O). Anal. Calcd. for C_22_H_23_N_3_O_6_: C, 62.11; H, 5.45; N, 9.88. Found: C, 61.37; H, 5.67; N, 10.04.

*Methyl 6-(4-(2-(2-acetamidophenyl)-2-oxoacetamido)benzamido)hexanoate* (**4h**). White powder, mp: 180–182 °C, yield 83%. ^1^H-NMR (DMSO-*d_6_*) δ (ppm): 1.32–1.36 (m, 2H, NHCH_2_CH_2_CH_2_CH_2_CH_2_CO), 1.49–1.56 (m, 4H, NHCH_2_CH_2_CH_2_CH_2_CH_2_CO), 2.00 (s, 3H, COCH_3_), 2.31 (t, *J* = 7.32 Hz, 2H, NHCH_2_CH_2_CH_2_CH_2_CH_2_CO), 3.25 (t, *J* = 5.88 Hz, 2H, NHCH_2_CH_2_CH_2_ CH_2 _CH_2_CO), 3.63 (s, 3H, COOCH_3_), 7.28 (t, *J* = 5.2 Hz, 1H), 7.64–7.85 (m, 7H, Ar), 8.38 (brs, 1H, NH), 10.55 (s, 1H, NH), 10.88 (s, 1H, NH); ^13^C-NMR (DMSO-*d_6_*) δ (ppm): 24.76 (COCH_3_), 26.5 (NHCH_2_CH_2_CH_2_CH_2_CH_2_CO), 29.4 (NHCH_2_CH_2_CH_2_CH_2_CH_2_CO, 33.8 (NHCH_2_CH_2_CH_2_CH_2_CH_2_CO), 41.5 (NHCH_2_CH_2_CH_2_CH_2_CH_2_CO), 51.7 (COOCH_3_), 119.9, 122.4, 124.4, 126.5, 128.5, 130.8, 131.6, 134.3, 138.2, 140.9, 162.3 (CONH), 166.0 (CONH), 169.5 (CONH), 173.9 (COOCH_3_), 189.7(COCO). IR (cm^−1^): 3290 (NH), 1738, 1664, 1637, 1608, 1528 (C=O). Anal. Calcd. for C_24_H_27_N_3_O_6_: C, 63.56; H, 6.00; N, 9.27. Found: C, 63.38; H, 6.13; N, 9.53.

*Methyl 1-(4-(2-(2-acetamidophenyl)-2-oxoacetamido)benzamido)cyclopentanecarboxylate* (**4i**). White powder, mp: 238–240 °C, yield 78%. ^1^H-NMR (DMSO-*d_6_*) δ (ppm): 1.69–1.71 (m, 4H, CH_2_CH_2_CH_2_CH_2_), 1.99 (s, 3H, COCH_3_), 2.00–2.13 (m, 4H, CH_2_CH_2_CH_2_CH_2_), 3.58 (s, 3H, COOCH_3_), 7.28 (t, *J* = 6.16 Hz, 1H,), 7.63–7.87 (m, 7H, Ar), 8.50(s, 1H, NH), 10.55(s, 1H, NH), 10.90 (s, 1H, NH); ^13^C-NMR (DMSO-*d_6_*) δ (ppm): 24.1 (CH_2_CH_2_CH_2_CH_2_), 24.7 (COCH_3_), 39.8(CH_2_CH_2_CH_2_CH_2_), 52.5 (COOCH_3_), 66.2 (C), 119.8, 122.5, 124.5, 125.9, 129.0, 130.0, 131.5, 134.2, 138.0, 141.3, 162.2 (CONH), 166.4 (CONH), 169.5 (CONH), 174.9 (COOCH_3_), 189.5 (COCO). IR (cm^−1^): 3291, 3114 (NH), 1746, 1679, 1633, 1607, 1524 (C=O). Anal. Calcd. for C_24_H_25_N_3_O_6_ (451.47): C, 63.85; H, 5.58; N, 9.31. Found: C, 64.06; H, 5.65; N, 9.04.

## 4. Conclusions

In conclusion, we have demonstrated that reaction of *N-*acetylisatin (**1**) with 4-aminobenzoic acid (**2**), either using conventional heating or microwave irradiation affords the α-ketoamide 4-(2-(2-acetamidophenyl)-2-oxoacetamido)benzoic acid (**3**) in good yield. All the spectral data proved the ring opening structure in which the 4-aminobenzoic acid attacks C2 rather than C3. Reaction of the 4-aminobenzoic acid derivative **3** with different amino acid esters using Oxyma/DIC afforded a novel series of 4-[2-(2-acetylaminophenyl)-2-oxo-acetylamino]benzoyl amino acid esters **4a**–**i**. OxymaPure/DIC showed clear superiority to HOBt/DIC and carbodiimide alone in terms of yield and purity.

## Supplementary Materials

Supplementary materials can be accessed at: http://www.mdpi.com/1420-3049/18/12/14747/s1.
