# Supplementary material for: OxymaPure/DIC: An Efficient Reagent for the Synthesis of a Novel Series of 4-[2-(2-Acetylaminophenyl)-2-oxo-acetylamino] Benzoyl Amino Acid Ester Derivatives
Source: Molecules. 2013 Nov 28;18(12):14747–59. doi: 10.3390/molecules181214747 (PMC6269765; doi:10.3390/molecules181214747)

Supplementary Materials

Figure S1. ^{1}H-NMR and ^{13}C-NMR of Compound **3**.

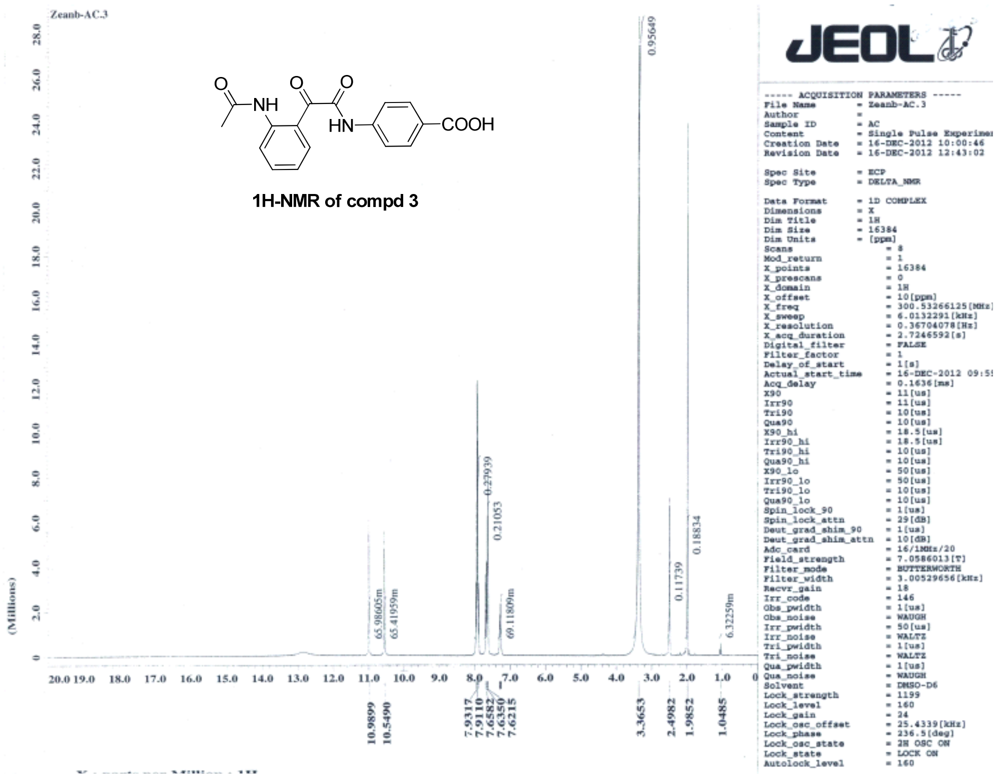

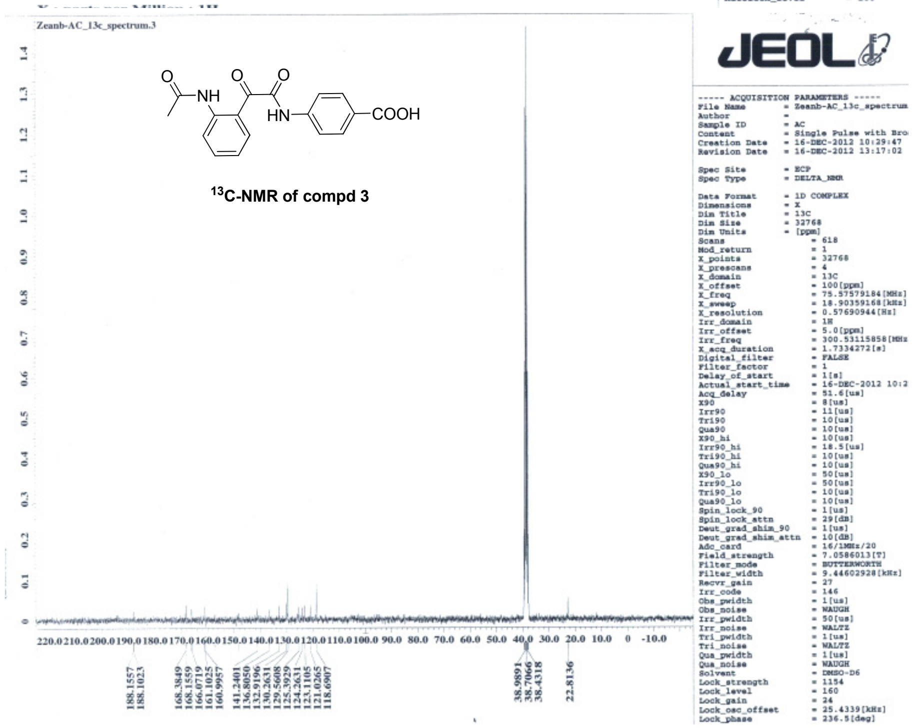

Figure S2. ^{1}H-NMR and ^{13}C-NMR of Compound **4a**.

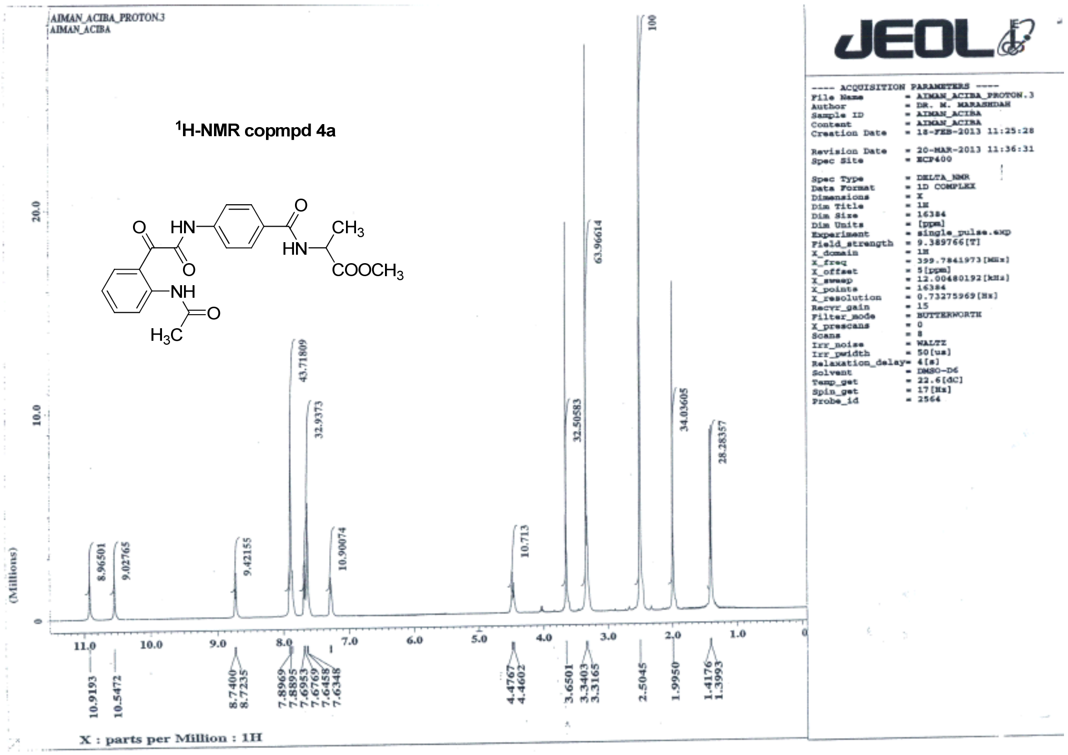

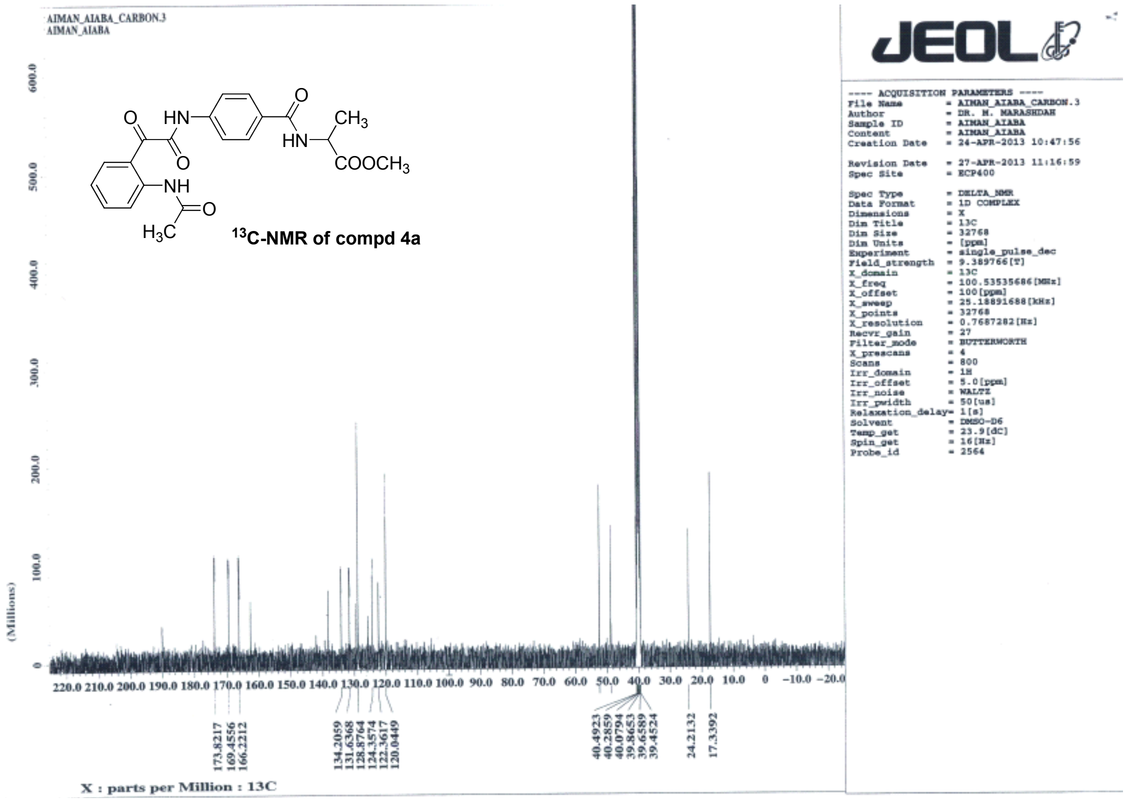

Figure S3. ^{1}H-NMR and ^{13}C-NMR of Compound **4b**.

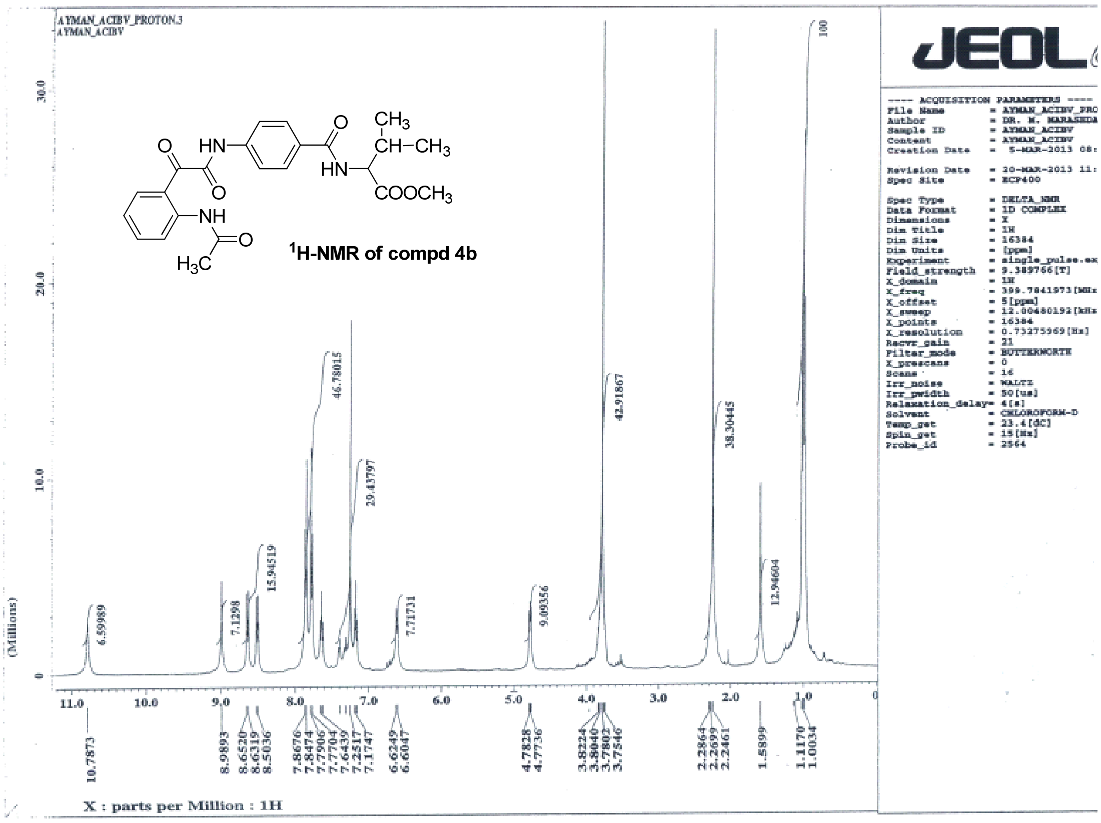

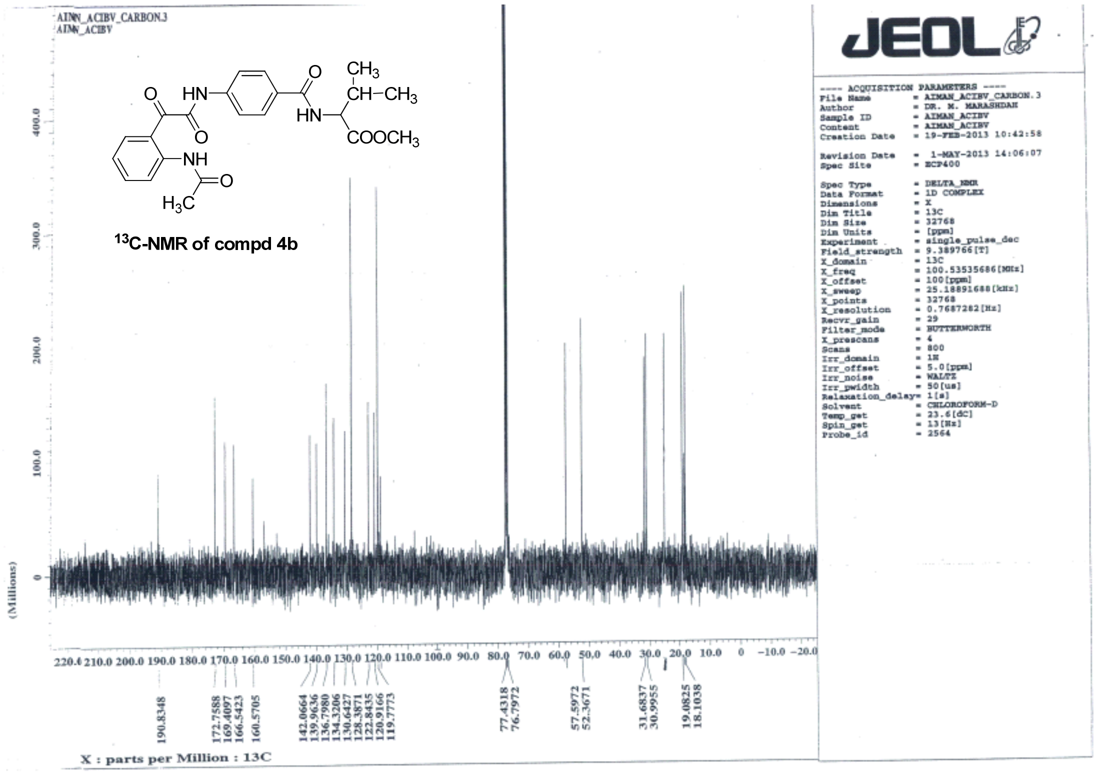

Figure S4. ^{1}H-NMR and ^{13}C-NMR of Compound **4c**.

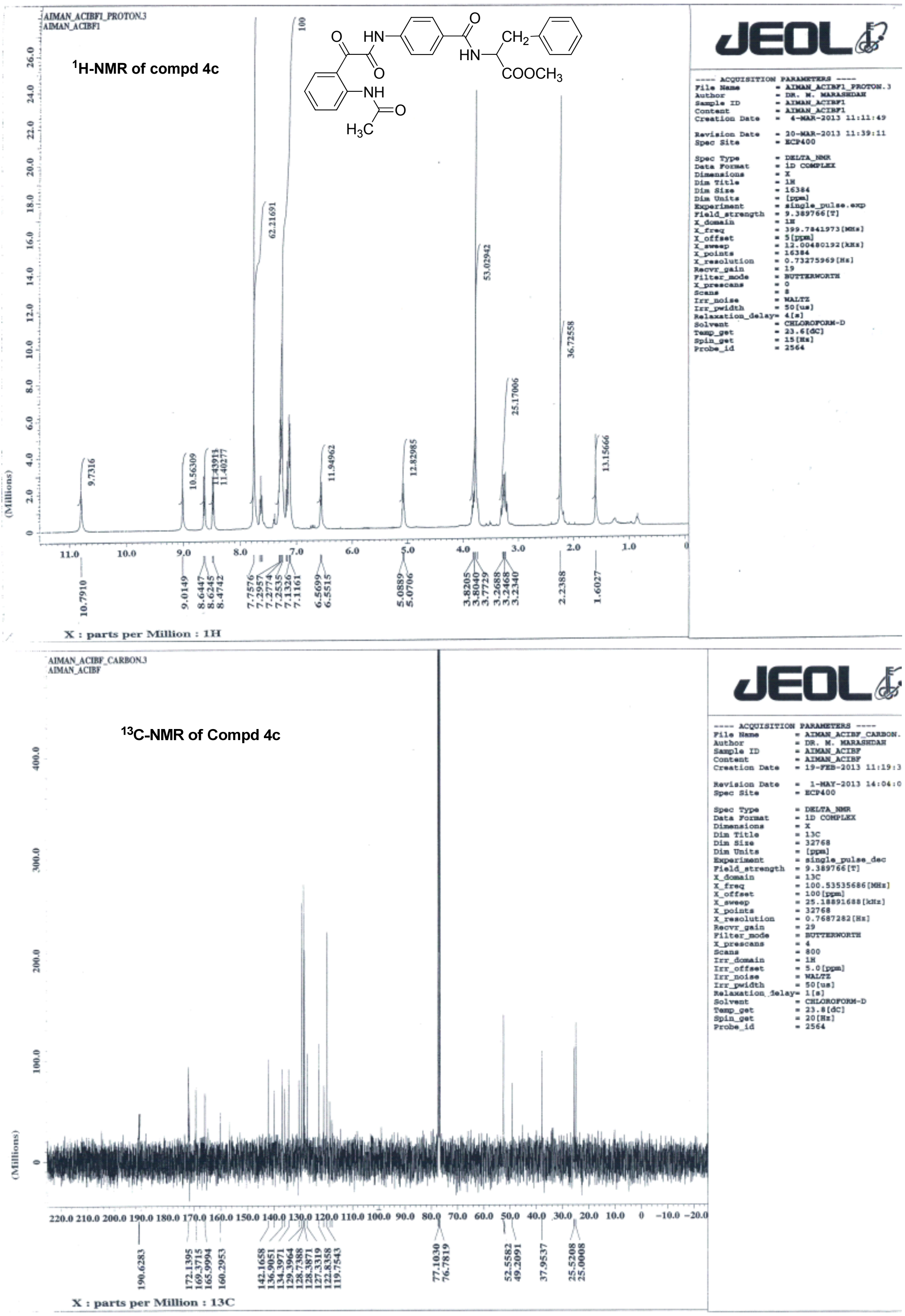

Figure S5. ^{1}H-NMR and ^{13}C-NMR of Compound **4d**.

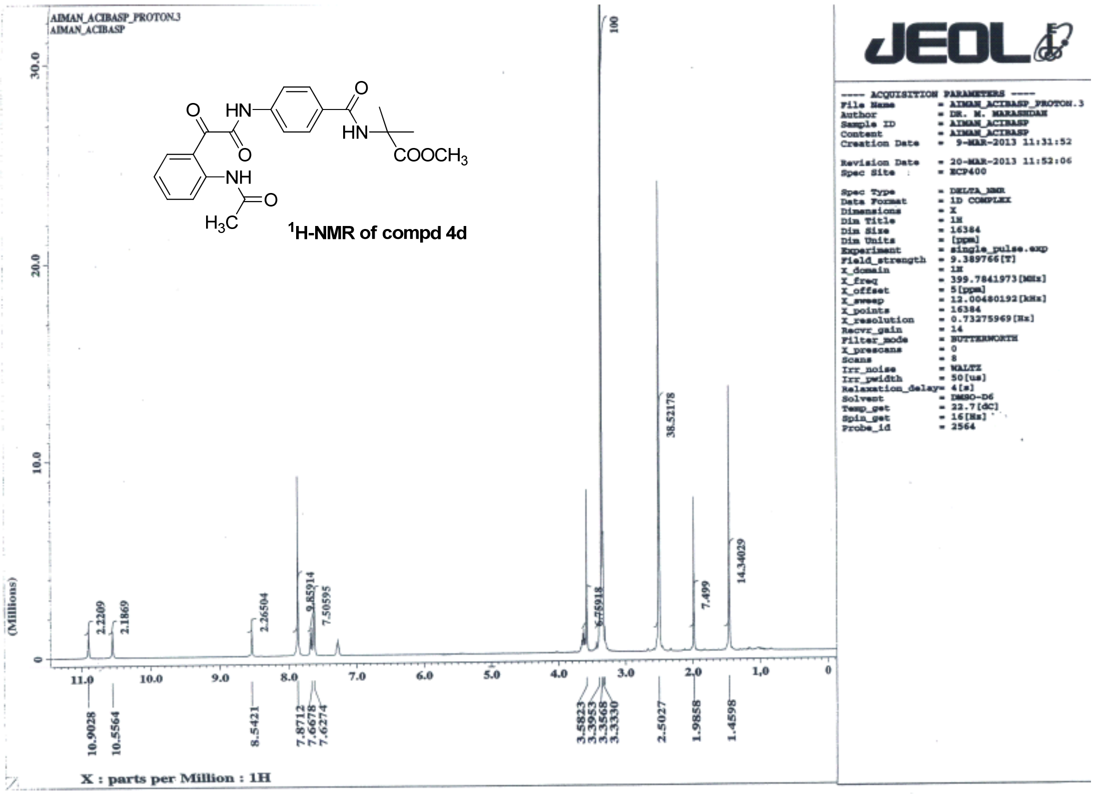

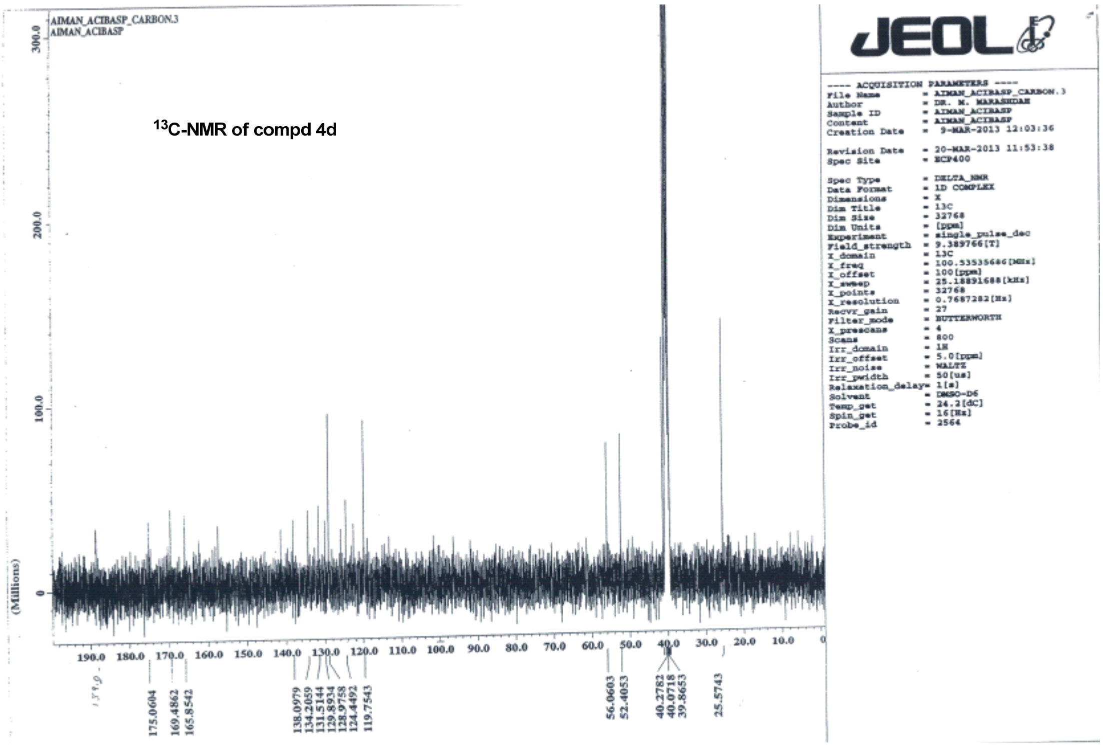

Figure S6. ^{1}H-NMR and ^{13}C-NMR of Compound **4e**.

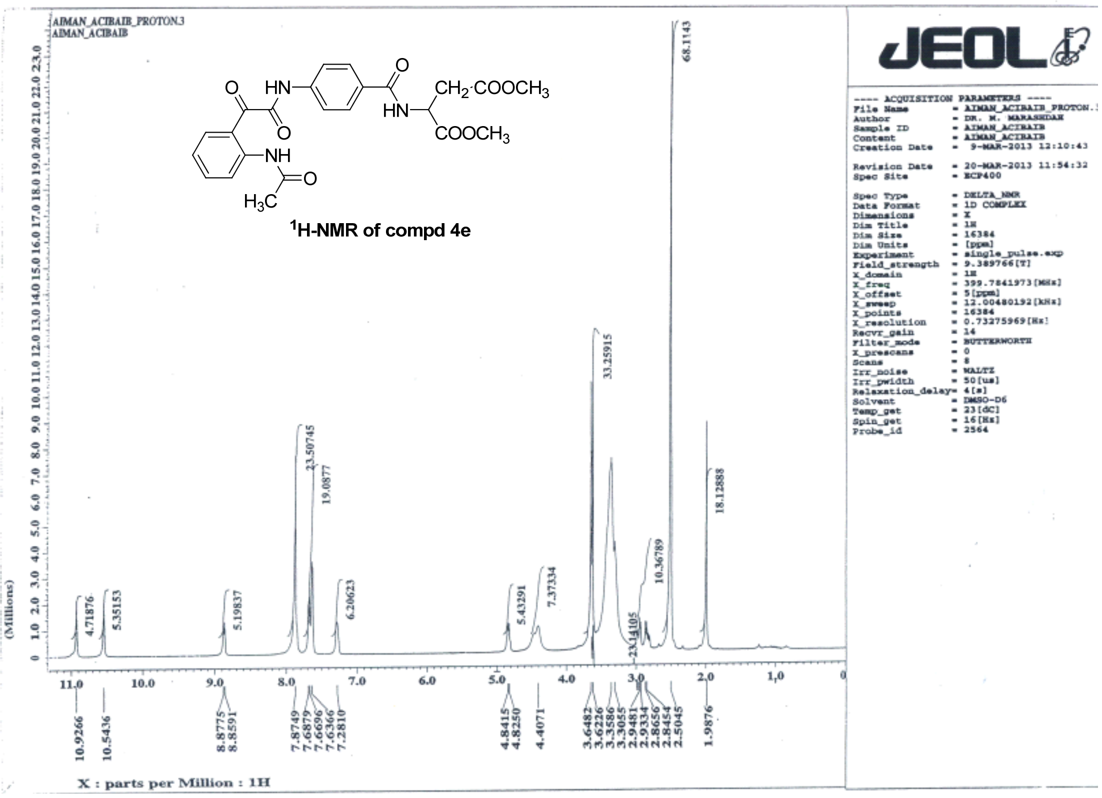

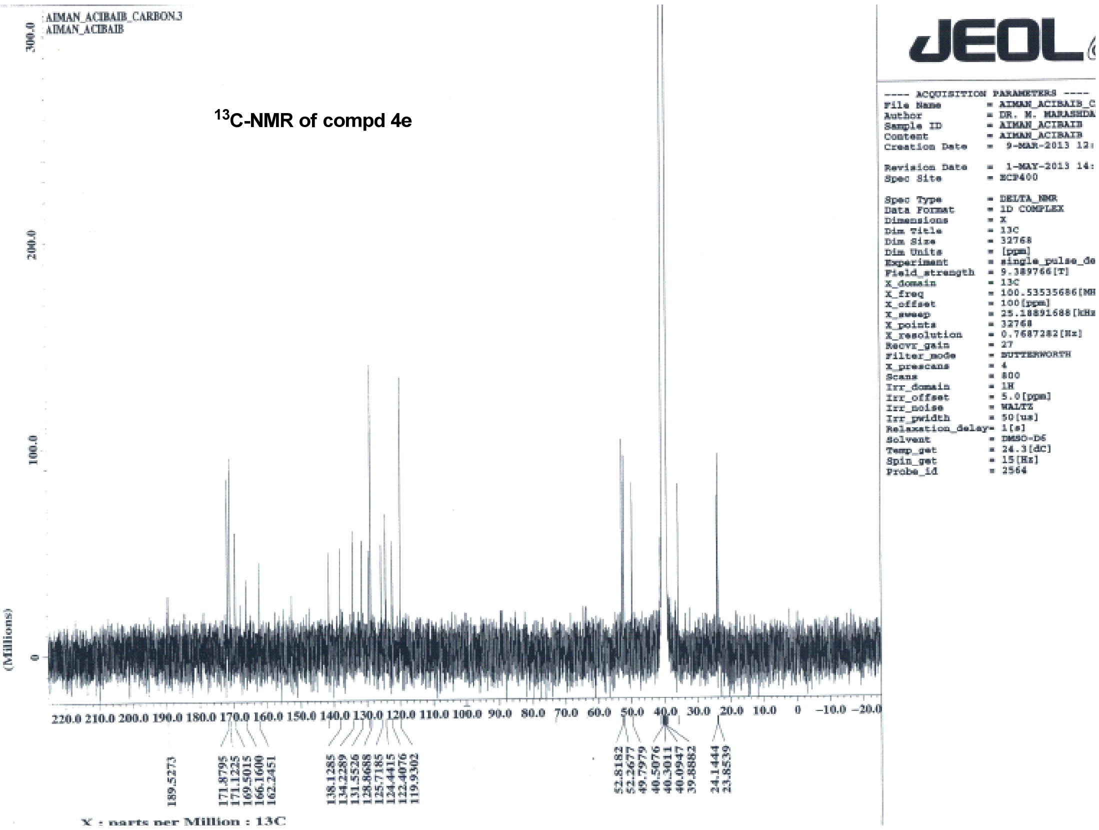

Figure S7. ^{1}H-NMR and ^{13}C-NMR of Compound **4f**.

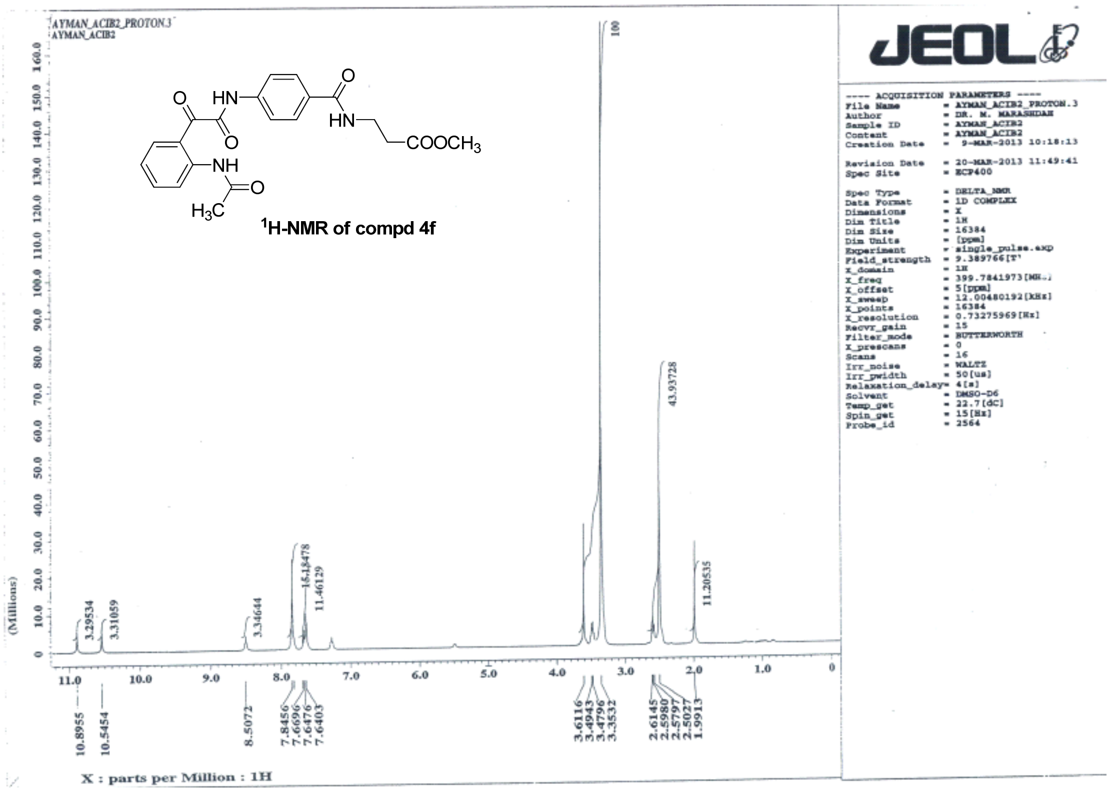

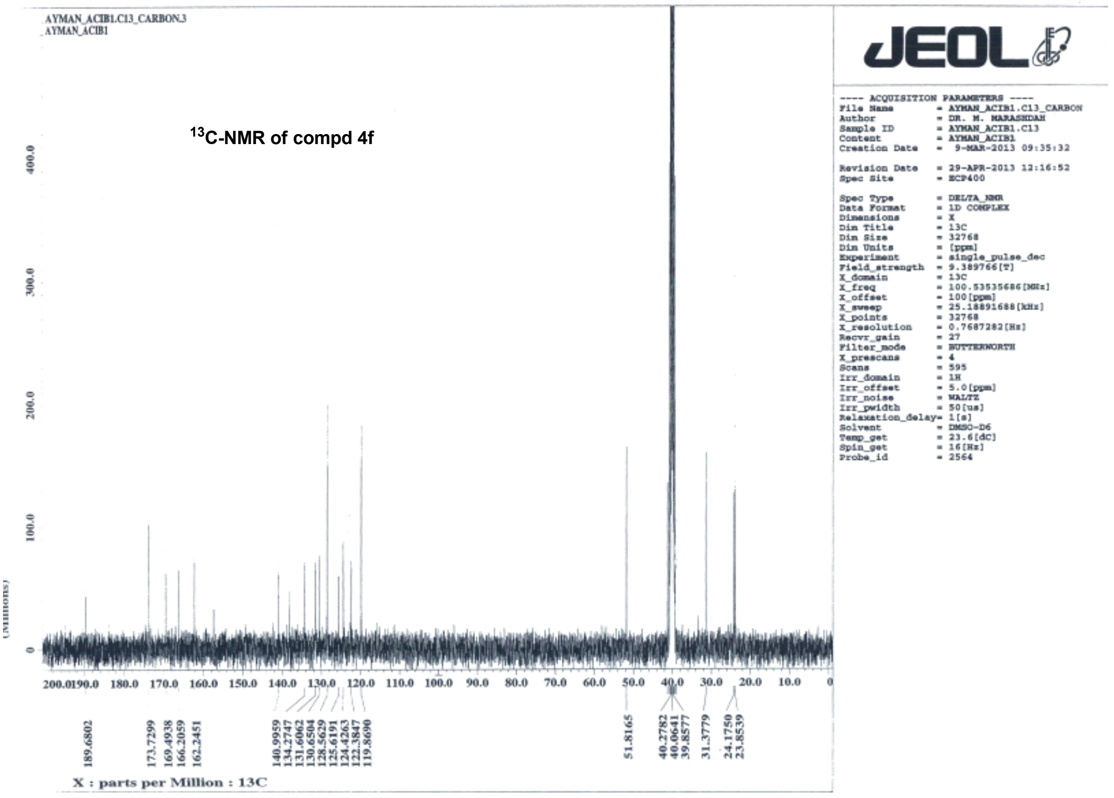

Figure S8. ^{1}H-NMR and ^{13}C-NMR of Compound **4g**.

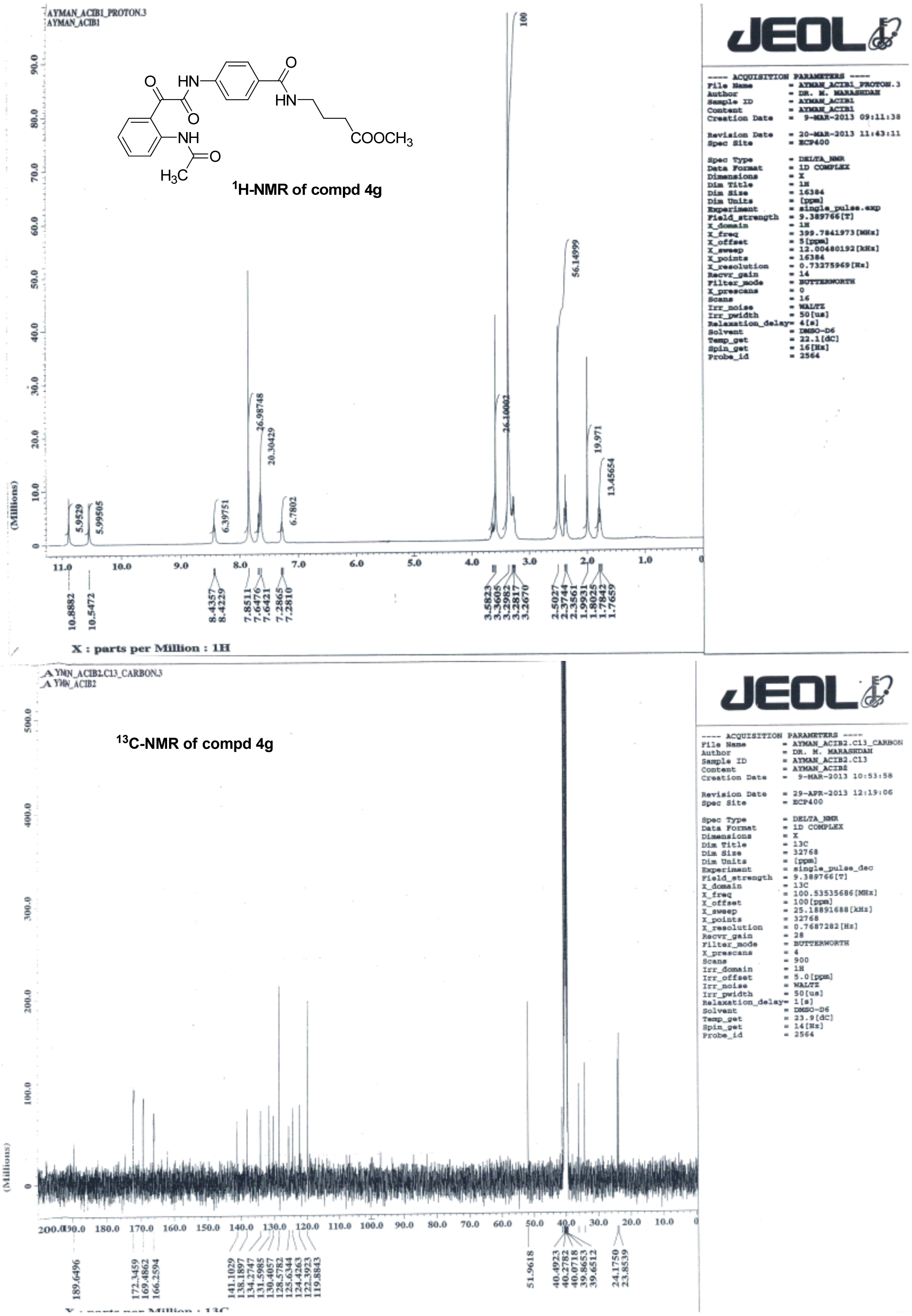

Figure S9. ^{1}H-NMR and ^{13}C-NMR of Compound **4h**.

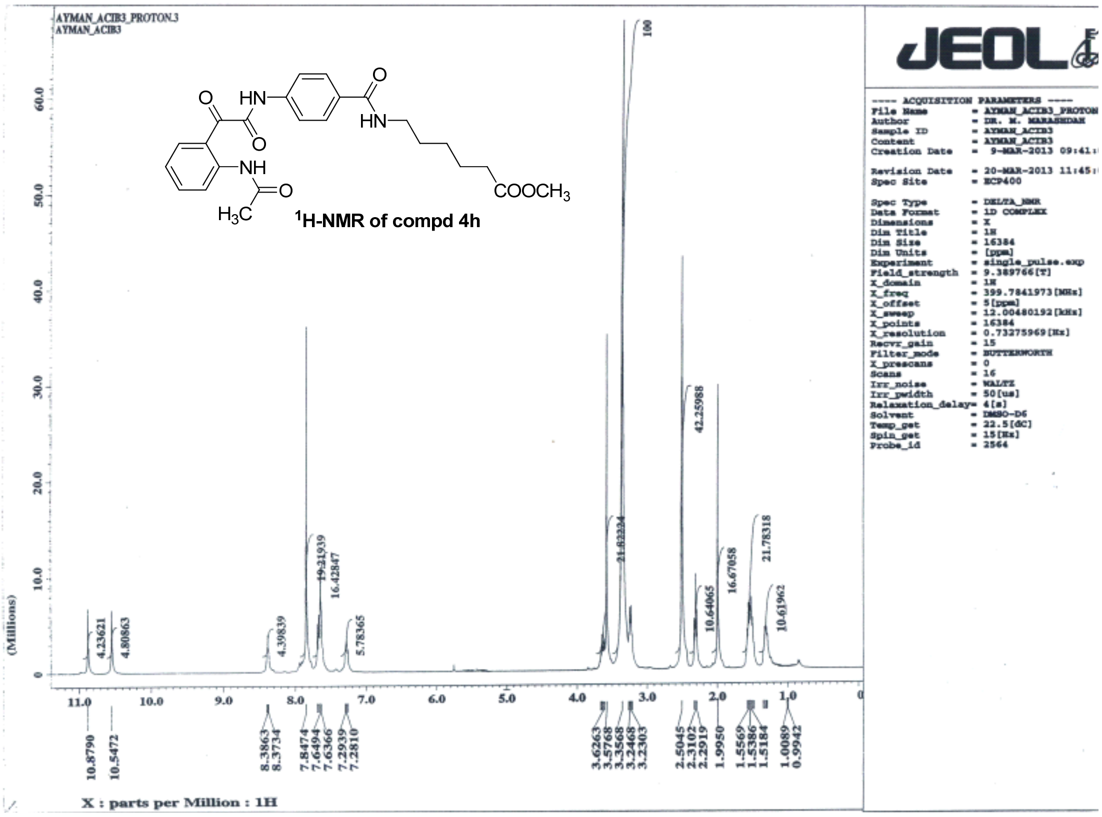

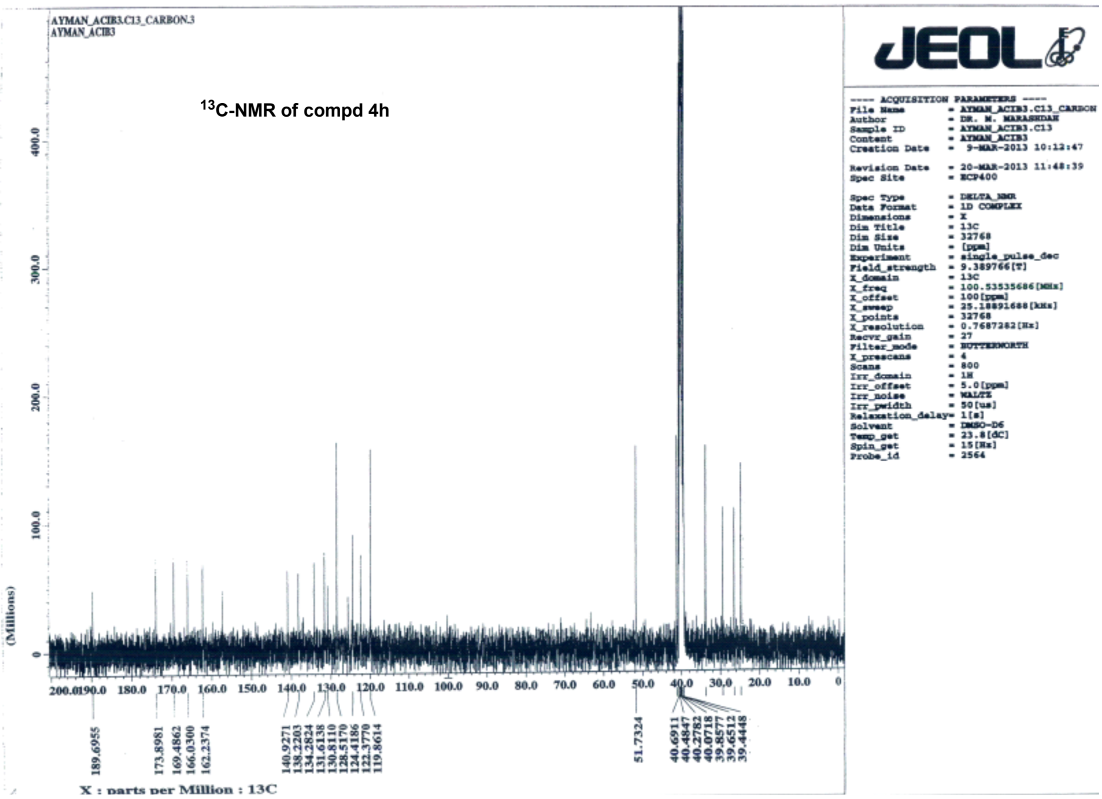

Figure S10. ^{1}H-NMR and ^{13}C-NMR of Compound **4i**.

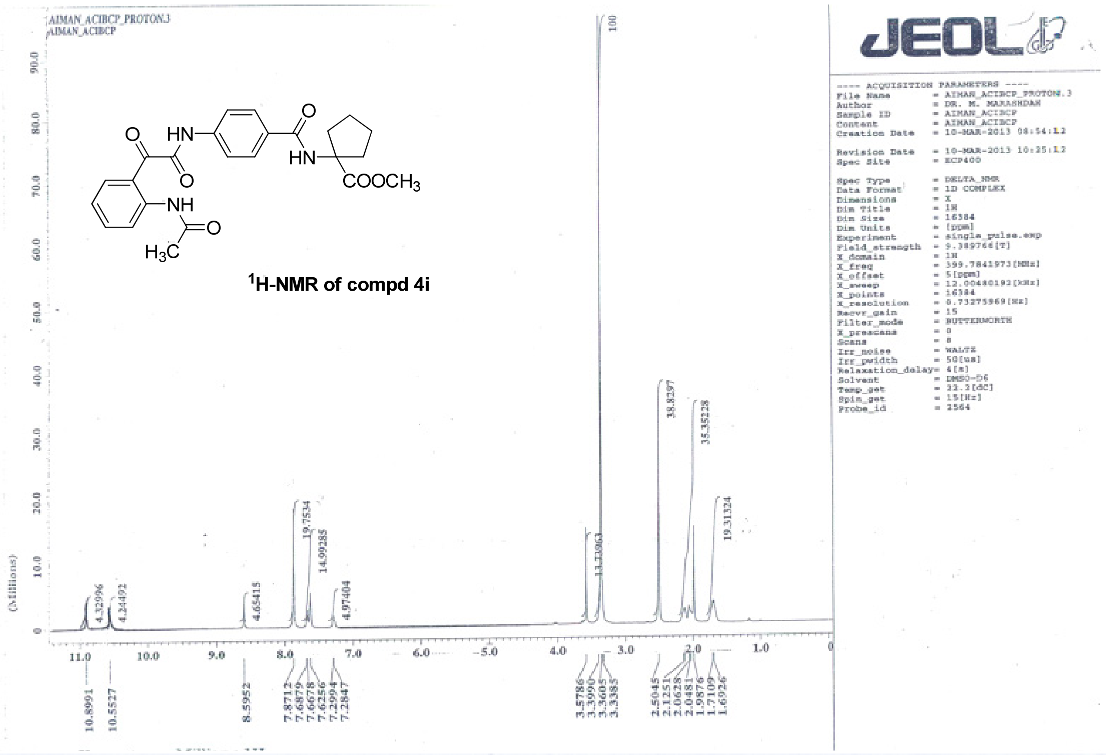

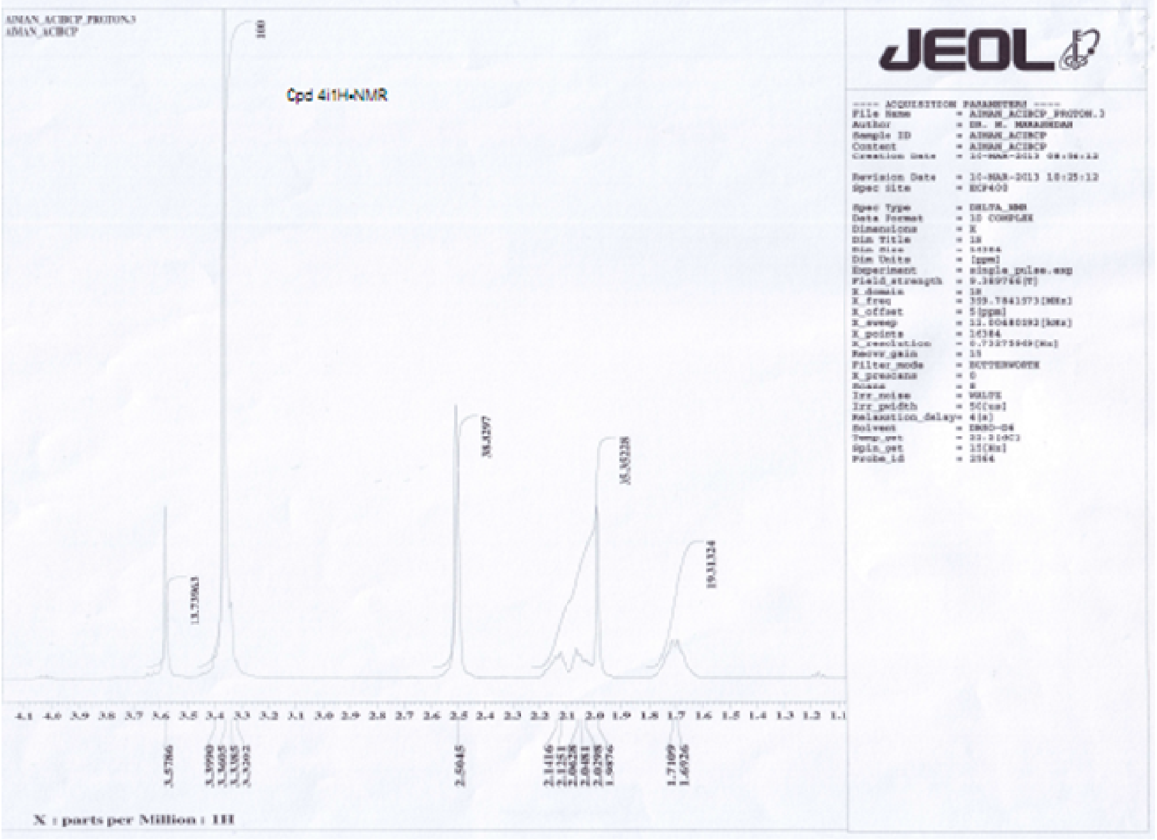

Figure 10. *Cont.*

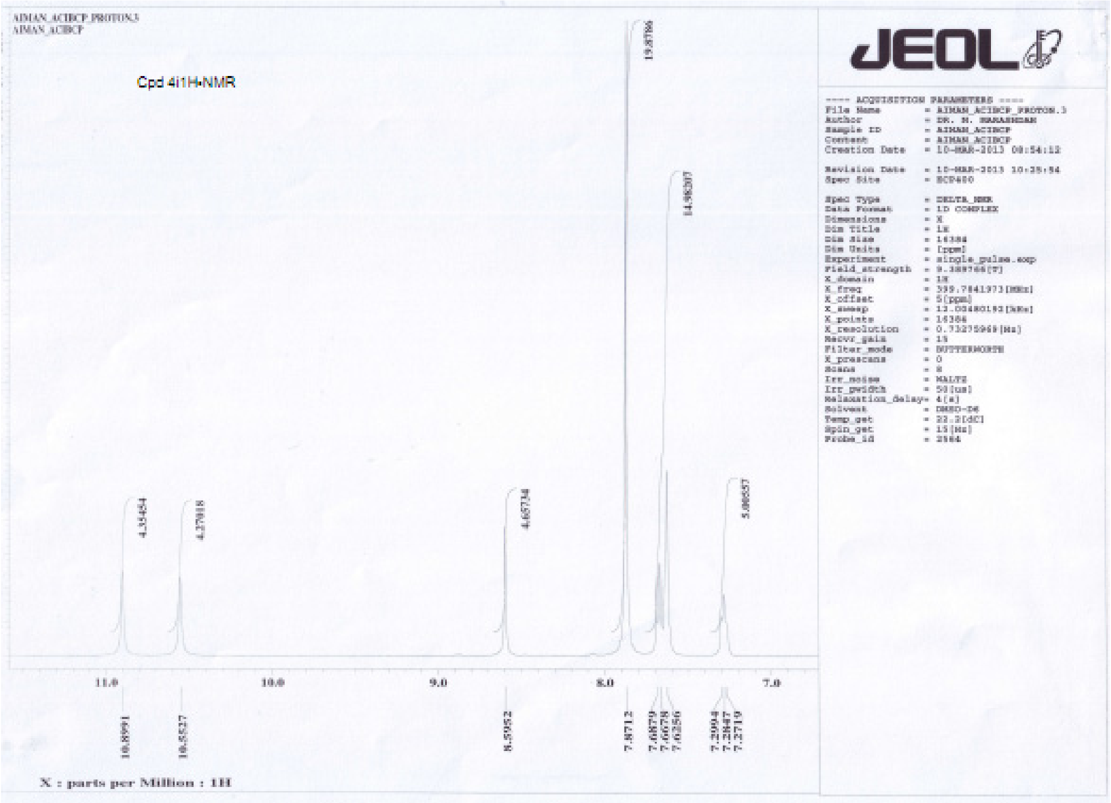

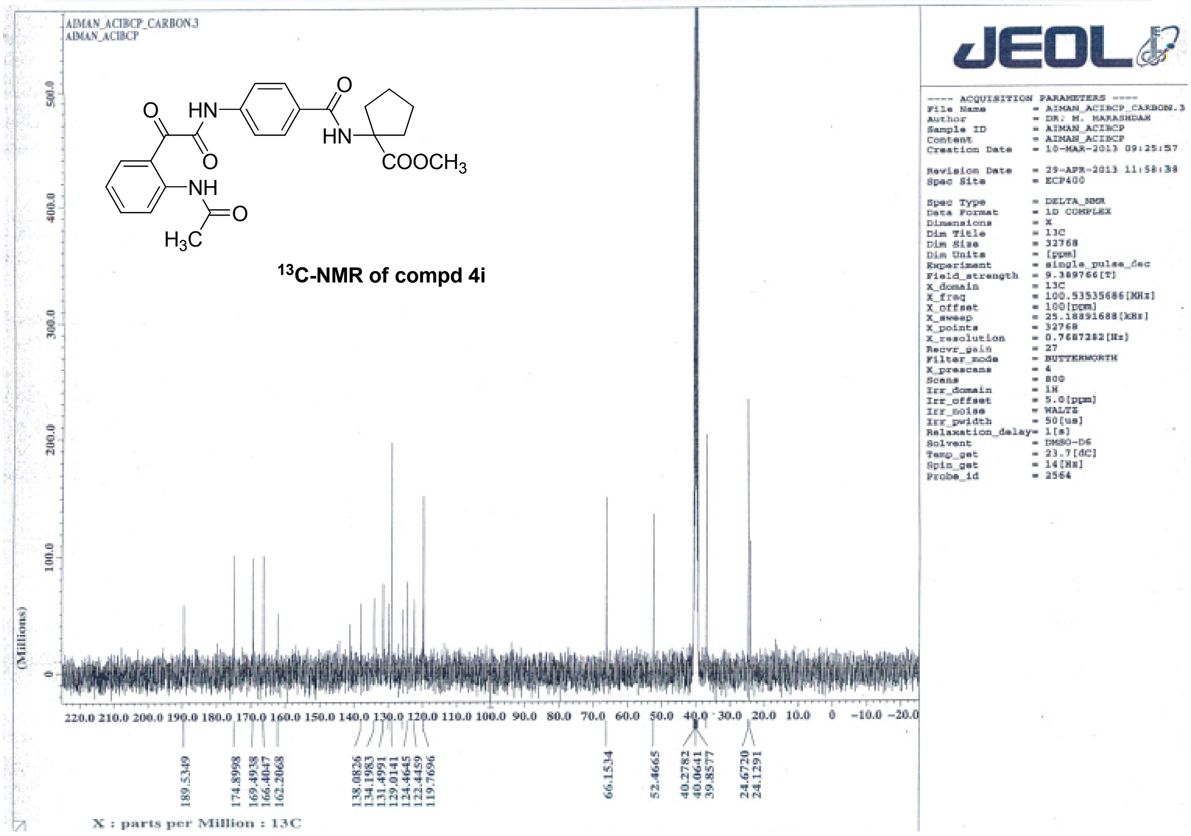

Supplement: Supplementary file 1 [file molecules-18-14747-s001.pdf]
